# CYCLICAL MIGRATION IN ALASKA NATIVE ELDERS AND ITS IMPACT ON ELDERS’ IDENTITY AND LATER LIFE WELL-BEING

**DOI:** 10.1093/geroni/igad104.0512

**Published:** 2023-12-21

**Authors:** Jordan Lewis, Steffi Kim, Zayla Asquith-Heinz, Lena Thompson

**Affiliations:** University of Minnesota Medical School, Duluth campus, Duluth, Minnesota, United States; University of Alaska Anchorage, Anchorage, Alaska, United States; University of Minnesota Duluth, Duluth, Minnesota, United States; University of Minnesota, Duluth, Minnesota, United States

## Abstract

This paper outlines a unique culturally driven cyclical migration of Alaska Native Elders. This Indigenous cyclical migration is distinct from other previously described mobility observations in that Elders spend extended time in more than one community. We describe the cyclical migration of Alaska Native (AN) Elders and its influence on the Elders’ identity, health, and well-being. Employing a life course perspective and social theory of migration, inductive content analysis was employed to identify themes related to Elders’ cyclical migration between rural and urban communities and the impact on their identity, health, and well-being. Interviews with 125 AN Elders were conducted across five regions of Alaska: Bristol Bay, Interior, Norton Sound, Aleutian Pribilof Islands, and Southcentral. AN Elders traveled between rural and urban communities to access resources and connections critical to their identities, health, and well-being. Urban Elders maintained a connection to rural villages because they perceive them as healthier places to age based on access to traditional practices, land, and the community. Rural Elders spent extended time in urban settings to access health care services, be closer to family, and to benefit from the lower costs of living. This study builds upon existing migration theories by introducing a cyclical pattern uniquely driven by AN identity, culture, and traditional practices. Findings illustrate how AN communities can support Elders who experience cyclical migration patterns to ensure they age successfully in both locations. Future recommended research should explore cyclical migration patterns among other Indigenous populations with histories of migration.

